# Deep Learning Predicts Underlying Features on Pathology Images with Therapeutic Relevance for Breast and Gastric Cancer

**DOI:** 10.3390/cancers12123687

**Published:** 2020-12-09

**Authors:** Renan Valieris, Lucas Amaro, Cynthia Aparecida Bueno de Toledo Osório, Adriana Passos Bueno, Rafael Andres Rosales Mitrowsky, Dirce Maria Carraro, Diana Noronha Nunes, Emmanuel Dias-Neto, Israel Tojal da Silva

**Affiliations:** 1 Laboratory of Computational Biology Bioinformatics, CIPE/A.C. Camargo Cancer Center, São Paulo 01508-010, Brazil; renan.valieris@accamargo.org.br (R.V.); lucas.amaro@hey.com (L.A.); adriana.passos@accamargo.org.br (A.P.B.); 2 Department of Pathology, CIPE/A.C. Camargo Cancer Center, São Paulo 01525-001, Brazil; cabtoledo@accamargo.org.br; 3 Department of Computation and Mathematics, University of São Paulo, Ribeirão Preto 14040-901, Brazil; rrosales@usp.br; 4 Laboratory of Genomics and Molecular Biology, CIPE/A.C. Camargo Cancer Center, São Paulo 01508-010, Brazil; dirce.carraro@accamargo.org.br; 5 Medical Genomics Laboratory, CIPE/A.C. Camargo Cancer Center, São Paulo 01525-001, Brazil; dnoronha@accamargo.org.br (D.N.N.); emmanuel@accamargo.org.br (E.D.-N.)

**Keywords:** digital pathology, deep learning, mutational signature, biomarker, DNA repair deficiency

## Abstract

**Simple Summary:**

DNA repair deficiency (DRD) is common in many cancers. This deficiency contributes to pathogenesis of the disease, but it also presents an opportunity for therapeutic targeting. However, current DRD identification assays are not available for all patients. We propose an efficient machine learning algorithm which can predict DRD from histopathological images. The utility of our method was shown by considering the detection of homologous recombination deficiency (HRD) and mismatch repair deficiency (MMRD) in breast and gastric cancer respectively. Our findings demonstrate that machine-learning approaches can be used in advanced applications to assist therapy decisions.

**Abstract:**

DNA repair deficiency (DRD) is an important driver of carcinogenesis and an efficient target for anti-tumor therapies to improve patient survival. Thus, detection of DRD in tumors is paramount. Currently, determination of DRD in tumors is dependent on wet-lab assays. Here we describe an efficient machine learning algorithm which can predict DRD from histopathological images. The utility of this algorithm is demonstrated with data obtained from 1445 cancer patients. Our method performs rather well when trained on breast cancer specimens with homologous recombination deficiency (HRD), AUC (area under curve) = 0.80. Results for an independent breast cancer cohort achieved an AUC = 0.70. The utility of our method was further shown by considering the detection of mismatch repair deficiency (MMRD) in gastric cancer, yielding an AUC = 0.81. Our results demonstrate the capacity of our learning-base system as a low-cost tool for DRD detection.

## 1. Introduction

DNA repair mechanisms have evolved to efficiently deal with various types of DNA damage from endogenous and/or exogenous sources. Independent of the source, however, DNA repair mechanisms are essential for maintaining genomic integrity in cells [1]. In certain human pathologies such as cancer, disruption of repair pathways lead to genomic instability which underlies the condition. Thus, repair pathways can also represent efficient targets for anti-tumor therapies [2]. In particular, the homologous recombination (HR) repair pathway plays a key role in maintaining genomic stability by driving high fidelity repair of double-stranded DNA breaks (DSBs) [3]. It has been documented that loss of function (LOF) mutations in genes involved in DSB repair via HR, such as *BRCA1* and *BRCA2*, confer breast and ovarian cancer susceptibility [4]. Correspondingly, tumors deficient in HR repair (HRD) are the most common forms of hereditary breast and ovarian cancer [4]. While HR-defective tumors have been found to be enriched in breast cancer cases, recent studies have demonstrated that other sporadic cancers share molecular and phenotypic characteristics of BRCA-mutant tumors, a concept termed as “BRCAness” [4,5]. An ability to stratify these tumors would have significant implications for cancer risk, treatment strategies, and patient survival [2,3,4,5,6].

Currently, biomarkers for HRD are used to identify tumors with events beyond germline or somatic LOF mutations in *BRCA1* or *BRCA2* [7]. Biomarkers for HRD are also used to identify tumors which carry additional genes involved in HR repair (HRR), such as somatic mutational signatures, gene expression profiles of genes which play crucial roles in tumor development, functional assays of protein expression, and “genomic scar” assays which use array-based comparative genomic hybridization. Due to the intricacy of the HR pathway, a combination of data regarding determinant molecular changes can improve the detection of tumors with HRD [7,8].

Comprehensive analyses of tumor-derived genome sequences have shown that a mutational signature known as Signature 3 (COSMIC database) is a promising biomarker for HRD in breast cancer [8,9]. Signature 3 is also recognized as a reliable readout of HR status. This signature arises from broad molecular changes (e.g., germline variants and somatic mutations in genes involved in HRR and epigenetic silencing of *BRCA1* and *RAD51c*, among others) in components of the HR pathway which are independent of BRCA1/2 mutations [8]. It has been proposed that HRD tumors can be stratified according to their Signature 3 profile, and this profile is more robust than specific assays which detect an isolated molecular change related to the HR pathway. However, despite being a promising biomarker of HRD, obtaining a Signature 3 profile is dependent on the availability of next-generation sequencing. Consequently, this profiling approach is not widely available in all clinical settings. As a result, potential opportunities to identify patients who may benefit from poly (ADP-ribose) polymerase inhibitor treatments have been limited [10]. Indeed, not all patients are screened for HRD status at the time of their cancer diagnosis. Hence, efforts to uncover new approaches for quantifying HRD would be beneficial, and could also expand the therapeutic potential of HRD-targeting agents across many tumor types [7]. Development of a low-cost strategy to accurately and rapidly assess HRD status in any tumor sample is of particular interest.

To date, remarkable advances have been made in predicting the relationship between a cell’s genome and its phenotype, thereby facilitating personalized treatment strategies [11,12,13,14,15]. Most notably, the development of deep learning techniques to predict outcomes, disease recurrence, and to distinguish cancer type, tumor composition, gene mutations, and grading status are of great interest. Herein, we trained a deep-learning model on digital histopathological images to detect HRD tumors (Section 2, and further discussed in Section 3). The ground truth labels for the deep-learning process are defined according to the activity of underlying mutational signatures. These signatures represent a reliable readout of HRD. We demonstrate that histopathological images can provide an accurate and efficient predictor of HRD status. The machine learning techniques we used employ a novel end-to-end framework which is based on deep multiple instance learning (MIL), see Section 4. After demonstrating its feasibility in classifying tumors as HRD, we further explore the utility and performance of our method by applying it to a cohort of patients with gastric cancer. We detected mismatch repair deficiency (MMRD), a repair pathway which is frequently impaired in many cancers [16]. Extensive evidence has also proved MMRD to be a reliable predictive biomarker guiding the treatment of some cancers [16,17]. Our pre-trained models are publicly available and can be successfully adapted to other specific types of DNA repair deficiencies, independent of a cancer’s tissue of origin. With immunotherapy being a focus of the cancer therapeutic paradigm in recent years, we anticipate that a low-cost, screening-based tool as described here would have great potential.

## 2. Results

### 2.1. Weakly Supervised Deep Learning for DNA Repair Deficiency (DRD) Assessment

**Figure 1:**
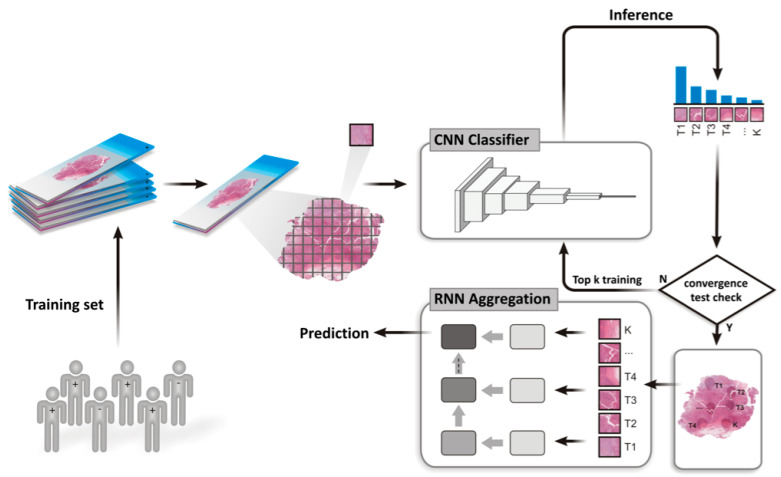
Overview of the end-to-end framework of training and testing for DNA repair assessment on whole slide images.

**Figure 2:**
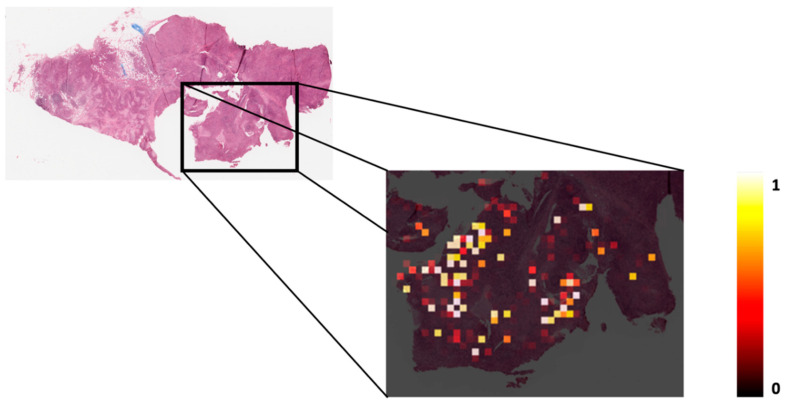
A whole slide image (32x) and corresponding heatmap for a single patient in the independent test set. The intensity of coloring in the heatmap represents the probability of being class-positive in the CNN (convolutional neural network) step.

Here, we describe a deep learning model for DRD (DNA repair deficiency) assessment which is applied to hematoxylin and eosin (H&E) stained histopathological whole slide images. Briefly, breast and gastric cancer samples with matched molecular data and histopathology images were collected from The Cancer Genome Atlas (TCGA). Next, the ground truth set of positive instances in the data set, which represent DNA repair deficient tumors, was defined according to mutational activity (see Methods). The architecture of our deep learning model is depicted in Figure 1, and it comprises the following steps. First, during the inference and learning steps, a whole slide image is tessellated into tiles. Otsu’s global thresholding is subsequently employed to discard all background tiles. The remaining tiles are then loaded into a convolutional neural network (CNN) in order to rank the tiles according to their probability of being positive (Figure 2). With learning gained from the top-ranking tiles per slide, a recurrent neural network (RNN) is used to establish a final slide classification according to slide-level aggregation. The latter process also takes into account the most promising tiles previously detected from the CNN.

**Figure 3:**
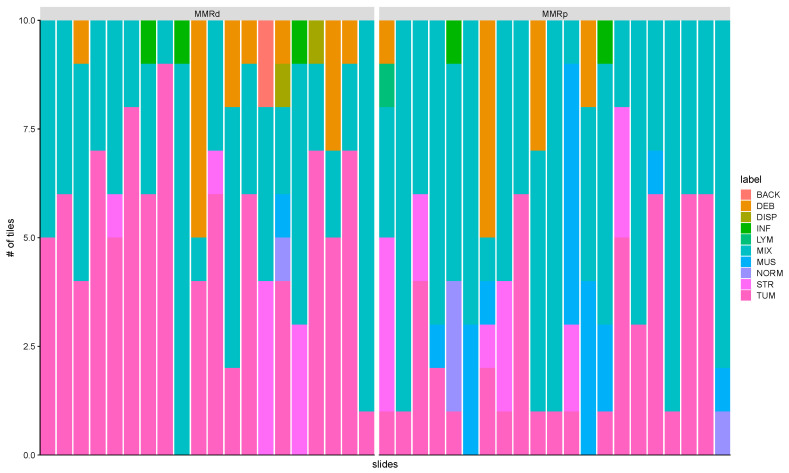
Tissue classes learned from convolutional neural network (CNN) in stomach cancer. Examples from mismatch repair deficiency (MMRD) and mismatch repair proficiency (MMRP) groups. The bar represents the spectrum of histologic diversity in the test set and it contains the top-ranking tiles per slide learned from CNN. These tiles were manually annotated by two pathologists among the following ten tissue labels: ADI, adipose tissue; BACK, background; DEB, debris; DIS, dysplasia; INF, inflammation; LYM, lymphocytes; MUS, smooth muscle; NORM, normal gastric mucosa; STR, cancer-associated stroma; TUM, gastric adenocarcinoma epithelium.

Notably, when tracking down the spatial patterns learned by the CNN while using an independent validation dataset, we noticed that our model selects regions which are composed of tiles that include several cell types (Figure 3 and Figure S1). This result suggests that tile-embedded cell diversity harbors underlying features which can predict DRD.

**Figure 4:**
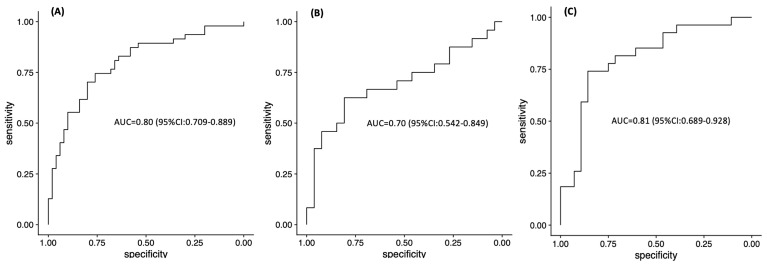
ROC analysis of classifier predicting DRD (DNA repair deficiency) status on (**A**) TCGA-BRCA training cohort, (**B**) ACCC-BRCA independent validation cohort and (**C**) TCGA-STAD training cohort.

Our model detected HR deficiency in breast tumors with an area under curve (AUC) of 0.80 (95% confidence interval (CI): 0.709–0.889, Figure 4A). When the model was challenged with an independent cohort of triple-negative breast cancer (TNBC) whole slide images taken from formalin-fixed paraffin embedded (FFPE) sections (65 slides from 65 patients), the AUC was 0.70 (95% CI: 0.542–0.849, Figure 4B). These breast cancers were previously characterized as proficient or deficient in HRR as described in the Methods section [18]. Taken together, these results demonstrate that our model is consistent.

Next, we tested the performance of our approach to predict MMRD in gastric cancer samples. For the cases analyzed, the AUC was 0.81 (95% CI: 0.689–0.928, Figure 4C). In addition, the MMRD prediction was consistent with a mutator phenotype known as microsatellite instability (MSI). The latter is characterized by an accumulation of insertions or deletions of small tandem DNA repeats, known as microsatellites [19], and high overall mutation rates. Our model was trained to predict mismatch repair (MMR) status, yet we observed that most of the patients (83%) who were predicted to have MMRD also harbored a MSI phenotype (see Section 2.3). This important result highlights the value of our approach in considering mutational signatures as a surrogate for MMRD during our training tests. Consequently, our model achieved a similar potential in predicting MSI status.

Taken together, our results demonstrate and substantiate the capacity for machine-learning methods to be used in advanced applications to support therapeutic decisions.

### 2.2. Mutational Signature as a Quantitative Label for DNA Damage Repair Status

While it is acknowledged that targeted genetic assays facilitate the classification of DRD tumors, comprehensive studies which have assessed exome, whole-genome, and sequencing panels have also revealed that mutational signatures are physiological readouts of DNA damage and DNA repair processes based on broader molecular changes in repair pathway components [8,9,20,21,22]. Therefore, we initially considered groups of samples with high versus low mutational signature activities for a given DNA repair pathway as a ground truth set for specific repair pathways. Mutational signatures were used since histomorphologic footprints are often preceded by harbinger DNA mutations which can be collectively identified in mutational signatures. Our results suggest that these mutational signatures are reflected in the morphological alterations detected by our approach. Moreover, the identification of signatures facilitates the development of a rapid and low-cost method which can be applied to a range of clinically relevant information involving tumor etiology.

### 2.3. Image Classification Recapitulates Unique Molecular Drivers

**Figure 5:**
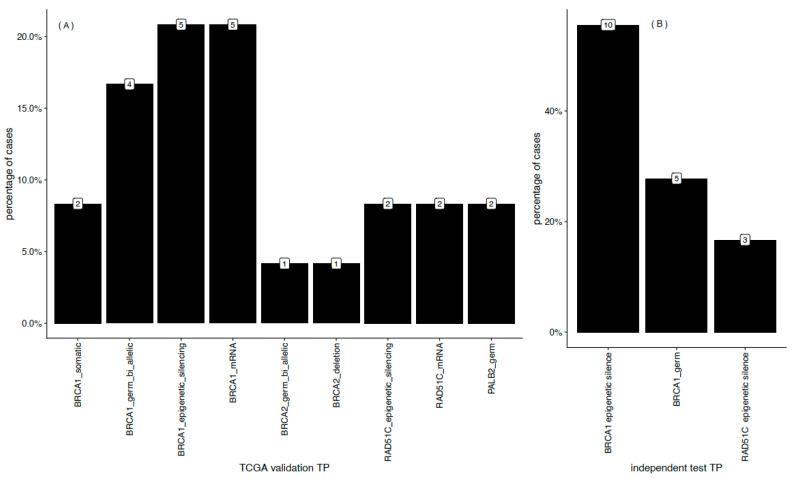
HR (homologous recombination)-associated molecular drivers. The distribution of lesions in known HR-pathway genes of the MMRD predicted patients among the training (**A**) and independent cohorts (**B**). Numbers above each bar count the patients for the corresponding lesion.

After demonstrating that our approach can accurately distinguish underlying features which are predictive of DNA repair status, we examined the molecular drivers involved. In this second stage, we took advantage of comprehensive alterations which are likely to contribute to HRD in breast tumors [9]. In Figure 5A, the proportion of molecular lesions are shown by type in selected genes of the HR pathway in the breast cancer cases examined [8]. Independent of variants in *BRCA1* (germline biallelic variants, epigenetic silencing, and somatic variants) and *BRCA2* (deletion and germline biallelic variants), other events were detected among HR pathway genes. For example, germline variants in *PALB2* and epigenetic silencing in *RAD51C* were detected. The latter is reflected by low expression levels. Consistent with these findings, our model also correctly identified patients with functional deficiencies incurred from inactivation of genes related to the HR pathway in an independent breast cancer cohort (Figure 5B). Moreover, the molecular profiles obtained are consistent with previous characterizations of HRD mechanisms [10].

**Figure 6:**
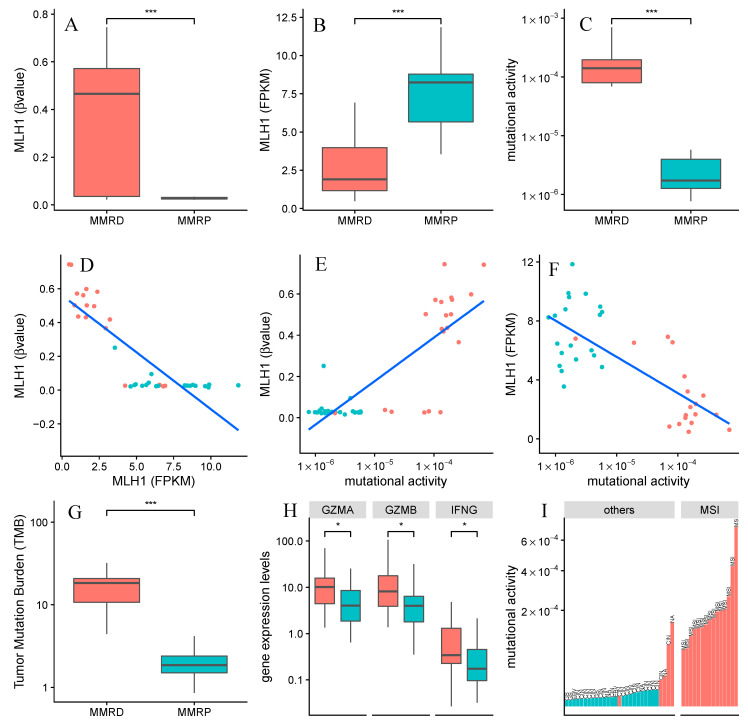
MMR (mismatch repair)-associated molecular drivers. This figure shows the molecular interplay among the MMRD and MMRP predicted groups, which are colored in red and blue, respectively. (**A**) Changes in methylation of *MLH1* promoter, (**B**) *MLH1* expression levels and (**C**) mutational activity of Signature 20 were detected by comparing MMRD and MMRP groups; Scatterplots showing (**D**) methylation and gene expression levels of *MLH1*, (**E**) methylation levels of *MLH1* and mutational activity of Signature 20, (**F**) gene expression levels of *MLH1* and mutational activity of Signature 20; (**G**) Changes of Tumor Mutation Burden and (**H**) genes related to pro-inflammatory markers across MMRD and MMRP groups. (**I**) Mutational activity of Signature 20 across MSI subtype and others molecular subtypes. Statistical significance was assessed by the two-tailed Wilcoxon Rank Sum and it is indicated by * *p* < 0.05, *** *p* < 0.001. The MSI (Microsatellite Instability) and other subtypes (GS: Genomically Stable; EBV: EBV-positive and CIN: Chromosomal INstability) were collected from The Cancer Genome Atlas (TCGA) study.

To evaluate the molecular landscape of gastric cancer patients predicted as MMRD and mismatch repair proficiency (MMRP) by our TCGA-based model, we first examined the absence of MHL1 with assays of promoter hypermethylation (Figure 6A) and transcriptional downregulation (Figure 6B). We observed a negative correlation between decreased expression of MLH1 and hypermethylation of its promoter (Figure 6D). Taken together, these results support the hypothesis that epigenetic silencing of *MLH1* functions as a repressive transcriptional signal. The commonly accepted association of MLH1 and MMR-deficient gastric cancers is also confirmed by these data [23,24,25]. We further evaluated how mutational activity is modulated in our MMRD and MMRP groups (Figure 6C,E,F). Our analysis revealed high mutational activity in MMRD patients, which is expected when activity of MLH1, a protein which plays a critical role in correcting replication errors, is low (Figure 6F). Consistent with previous reports, we also observed higher tumor mutation burden (TMB) in the MMRD group compared to the MMRP group (Figure 6G). Cells with a high degree of TMB correlate with a greater probability of abnormal protein expression which can give rise to mutation-derived antigens (neoantigens) and recruitment of tumor-infiltrating leukocytes. Significant differences were observed between MMRD and MMRP tumors in regard to immune-related gene expression, including genes related to pro-inflammatory markers (Figure 6H). Furthermore, our data support the findings of other studies where MMRD tumors have exhibited increased mutation burden in combination with upregulated immune-related signaling [19].

## 3. Discussion

We evaluated the feasibility of a weakly supervised deep learning system for assessing DRD from whole slide images of FFPE tissues. We show that our TCGA-derived models are able to detect HR deficiency in breast cancer (AUC = 0.80), and subsequently, we successfully applied the inferred model to a dataset of FFPE tissues from an independent cohort (AUC = 0.71). Our classifier was further applied to predictions of DNA MMR deficiency in gastric cancer patients (AUC = 0.81). These results show that our model works reasonably well while dealing with DRD. Further, our methods can be used to detect deficiencies more generally in a broader context of other repair deficient pathways.

Classification of patients according to mutational signatures is strongly consistent with well known molecular profiles of repair deficiency components [8,9,20,22]. To further confirm this, we characterized our findings with known genotype-phenotype relationships by integrating data regarding gene expression levels, methylation status, and tumor-infiltrating lymphocyte composition across our test sets. Our findings (Figure 5 and Figure 6) show that patients that are classified as having both HRD and MMRD, harbor the molecular determinants that drive these deficiencies.

Previous studies have demonstrated the potential of digital pathology analysis by using deep learning [13,14,15]. However, manual segmentation remains a bottleneck step in the analysis of whole slide images. As a result, the development and implementation of this approach in the clinic has been severely hampered. Here, we overcome this shortcoming by using MIL, a machine learning paradigm which includes a full pass through a dataset to detect relevant local patterns in images [26].

Similar to other studies which have used FFPE-based samples, improvements are needed to increase the performance of the methods described [12,13,14,15,27]. First, weakly supervised deep learning on whole slide images requires appropriate exclusion of artifacts such as tissue preparation, tissue folds, torn tissue, and pen marks. We addressed this issue by considering Otsu’s thresholding methods by extensive experimentation. However, it is possible these methods could be further optimized. Larger training cohorts could also improve classification performance if the network learned from a broader range of morphological variants. Furthermore, improvements in classification accuracy of independent patient cohorts with different staining protocols and whole slide scanners could prevent overfitting.

In conclusion, the deep learning system for DRD assessment described here is a low-cost, screening-based tool which holds extraordinary potential for identifying patients who may be eligible for either genetic counseling or immunotherapy. In addition, this screening tool could identify which patients would derive the most benefit from receiving cancer treatment as early as possible. Our results demonstrate and add strength to the belief that machine-learning methods can be used for advanced applications to assist therapy decisions.

## 4. Materials and Methods

Classification of whole slides was modeled as previously described [27]. Briefly, whole slides are split into tiles and each tile is classified with a multiple instance learning model. Next, tile-level classifications of each slide are aggregated by using a recurrent network resulting in a single classification for the whole-slide (Figure 1). The following two sections further describe these steps in some detail.

### 4.1. Slide Pre-Processing

Initially, the available set of histopathological slide images was randomly divided into a training set (70%) and a validation set (30%). Samples with multiple slides were kept together in their respective sets (details in Tables S1 and S2). Next, each slide was resized to 0.5 μm/pixel magnification and split into 224 × 224 pixel non-overlapping tiles with *libvips* [28] and openslide [29]. An *Otsu* thresholding algorithm [30] was calculated for each tile. Tiles with a threshold value < 0.1 were discarded. After image preprocessing, a total of 37,140 tiles (TCGA-BRCA, n = 32250; TCGA-STAD, n = 3940; ACCC-BRCA, *n* = 1950) were generated.

### 4.2. Model Definition and Training

Classification of a slide was modeled as a multiple instance binary classification problem [26] (Figure 1), where each slide is a bag of tiles. Positive bags must have at least **K** positive tiles, while negative bags must have all negative tiles. Given a convolutional model, **M1**, all tiles are classified and ranked according to a **M1** prediction. The top **K** tiles of each bag are used to train **M1**, and this process is repeated until **M1** predictions converge for a given dataset. The result is a model which can distinguish tiles that are relevant to the whole-slide label from the rest.

Training tiles were fed into a resnet34 model [31] pre-trained with the ImageNet dataset [32]. For every epoch, the inference of each tile is calculated and the top **K** tiles are used to train this model according to whole slide labels. Test accuracy is calculated by comparing the prediction of the top tile of each slide with whole slide labels. Classification of a whole slide is modeled with a RNN. RNNs have an internal state which can be used to aggregate a sequence of inputs into a single prediction.

To train the RNN model, **M2**, the best tile-classifier model of the first step (**M1**) is fixed and the top **K** tiles of each slide are fed into the **M1** model. After the intermediate learned features are extracted, these features are fed into **M2** to aggregate all of the features of the top **K** tiles and output a single prediction variable. **M2** is trained with the same dataset that was used in the first step.

The M2 RNN architecture takes a 512-vector as input, which matches the size of the intermediate features output of resnet34, and a state (“memory”) vector. This vector aggregates the features of previous tiles fed into the RNN. The input and the state vector is combined with ReLU activation step resulting in a new state output. The latter is finally fed into another fully connected layer that outputs a single prediction variable.

All of our models were built and trained with PyTorch (available at https://pytorch.org). The computational framework for DRD status, including all parameters used in this paper, are freely available and described at https://github.com/rvalieris/deepHE. All models were trained by using NVIDIA TESLA V100 GPUs provided by AWS (Amazon Web Services).

### 4.3. Datasets

Clinical and molecular information for cases of breast cancer (TCGA-BRCA, 1011 patients, 1075 slides) and stomach cancer (TCGA-STAD, 369 patients, 394 slides) were gathered from the Genomic Data Commons Data Portal (https://portal.gdc.cancer.gov/). H&E-stained histologic images derived from the TCGA-based cohort were retrieved from The Cancer Imaging Archive (TCIA) repository. As an external validation cohort, we retrospectively analyzed archived H&E-stained pathology slides obtained from breast cancer patients treated at the A.C. Camargo Cancer Center (ACCC-BRCA, 65 patients, 65 slides). The HRD status for this independent cohort validation set was assessed by considering pathogenic germline mutations and epigenetic silencing in HR-related genes that were previously detected and reported [18]. Briefly, TNBC samples were characterized as HR proficient or HR deficient according to the presence of *BRCA1/2* germline or somatic pathogenic mutations in DNA from leukocytes and tumors, or *BRCA1* epigenetic silencing analyses of tumor DNA, respectively [18]. Additionally, a *RAD51c* epigenetic silencing analysis of tumor DNA was performed (data not published). Both clinical and molecular data from the patient cohorts analyzed during this study are presented in Supplementary Table S1.

### 4.4. Biomarker for HRD and MMRD Status

A biomarker score for HR and MMR status was used. Briefly, a pattern of genome-wide mutations (mutational process) known as a mutational signature (https://cancer.sanger.ac.uk/cosmic/signatures_v2) was used. This mutational signature reflected the proficiency of multiple upstream components of repair pathways [8,22]. Mutational signature activities for breast cancer (TCGA-BRCA) and their lesions in HR genes were previously detected and described [8]. For stomach cancer (TCGA-STAD), all somatic single nucleotide variants (SNVs) (e.g., C > A, C > G, C > T, T > A, T > C, and T > G) were mapped onto trinucleotide sequences by including the 5′ and 3′ neighboring base-contexts. Next, the SNV spectrum with 96 types of trinucleotide mutations for all samples was loaded into signeR [33]. A projection analysis was performed and signature activity across the samples was inferred by using a MMR-associated COSMIC signature. Finally, we performed assignments of per-patient signature activity. Thus, samples with higher mutational signature activity for a given DNA repair pathway were labeled as a ground truth set for that pathway. We used Signature 3 and Signature 20 as readouts of HRD and MMRD for breast and stomach cancers, respectively.

### 4.5. Data Availability

The dataset analyzed in this study is described in our Methods and Supplementary Data. Further information regarding this dataset is available from the authors upon request.

## 5. Conclusions

Our study demonstrates the use of an AI-based workflow capable of detecting DRD-positive tumors directly from H&E-stained histologic images. The method is illustrated by considering the detection of homologous recombination deficiency and mismatch repair deficiency across 1445 cancer patients, including breast and gastric cancer samples. The reliability of the results is shown by evaluating the model in an independent dataset. The success of detecting DRD-status, suggests that deep-learning models can be used to assess other clinically relevant information, that can potentially enhance traditional pathology reports.

## Supplementary Materials

The following are available online at https://www.mdpi.com/2072-6694/12/12/3687/s1, Figure S1: Tissue classes learned from Convolutional Neural Network (CNN) in breast cancer.

## Abbreviations

DNA repair deficiency (DRD), microsatellite instability (MSI), homologous recombination (HR)
