# Supplementary material for: Deep Learning Predicts Underlying Features on Pathology Images with Therapeutic Relevance for Breast and Gastric Cancer
Source: Cancers (Basel). 2020 Dec 9;12(12):3687. doi: 10.3390/cancers12123687 (PMC7763049; doi:10.3390/cancers12123687)

Figure S1: **Tissue classes learned from Convolutional Neural Network (CNN) in breast cancer**. Examples from Homologous Recombination Deficiency (HRD) and Homologous Recombination Proficiency (HRP) groups. The bar represents the spectrum of histologic diversity in test set and its contains the top-ranking tiles per slide learned from CNN. These tiles were manually annotated by pathologist among the following eight tissue labels: ADI, adipose tissue; BACK, background; DEB, debris; INF, inflammation; MIX, mix; MUS, smooth muscle; NORM, STR, cancer-associated stroma; TUM, breast carcinoma.

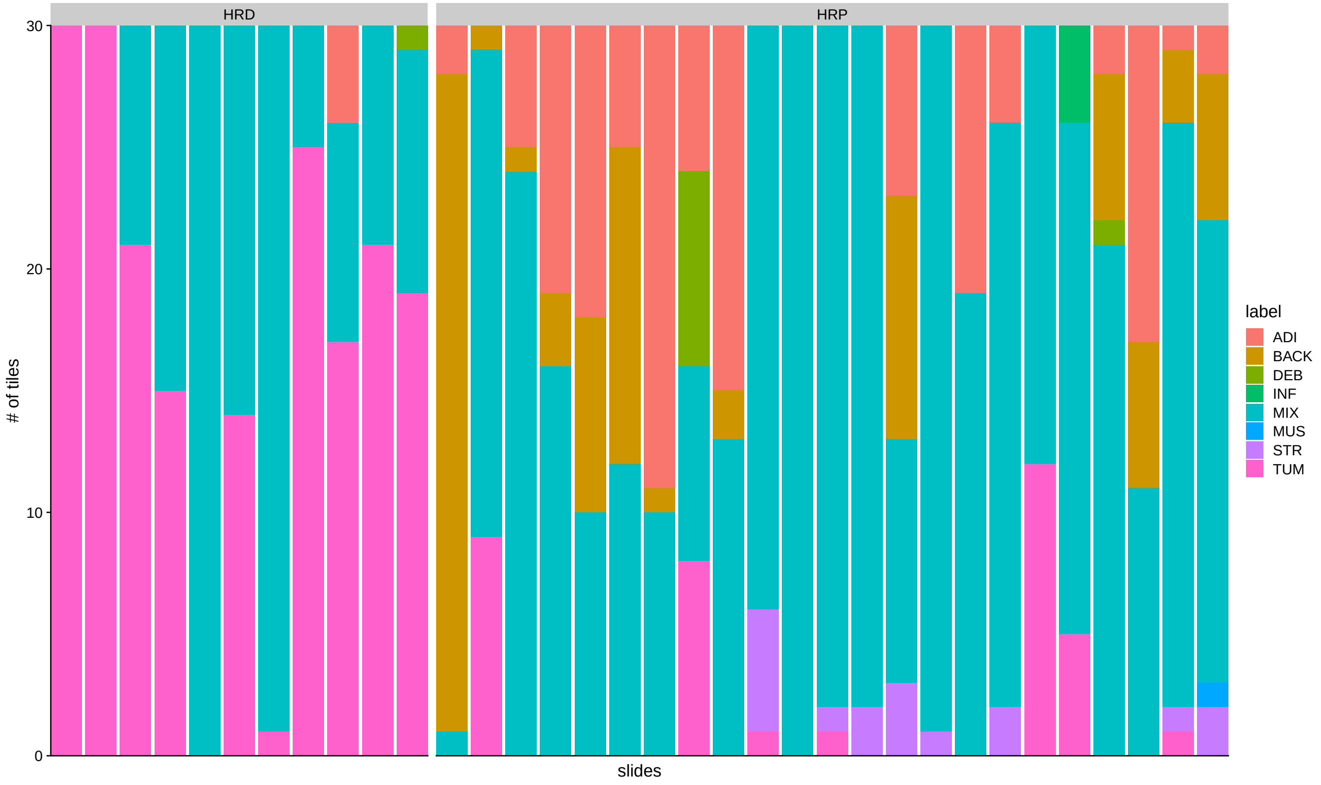

Supplement: Supplementary file 1 [file cancers-12-03687-s001.zip › supp.pdf]
